# Absence of the celiac trunk and anomalous very low origin of the common hepatic artery arising independently from the abdominal aorta just above aortic bifurcation in patient undergoing radical pancreaticoduodenectomy

**DOI:** 10.1007/s00276-020-02666-6

**Published:** 2021-01-15

**Authors:** Sławomir Mrowiec, Robert Król, Beata Jabłońska

**Affiliations:** 1 Department of Digestive Tract Surgery, Medical University of Silesia, Medyków 14 St. 40-752, Katowice, Poland; 2 Department of General, Vascular and Transplant Surgery, Medical University of Silesia, Katowice, Poland

**Keywords:** Celiac trunk, Common hepatic artery, Anatomic variation, Anomaly, Pancreaticoduodenectomy

## Abstract

**Purpose:**

Knowledge of anomalies of the celiac trunk is very important during various surgical procedures (such as pancreatic and gastric resections including Appleby operation, liver resections and liver transplantations) and as well as radiologic procedures (such as chemoembolization of pancreatic and hepatic tumors).

**Methods:**

A 77-years-old woman was admitted to our department for surgical treatment of ampullary adenocarcinoma G2 confirmed in endoscopic retrograde cholangiopancreatography (ERCP) with papillotomy and ampullary biopsy. In the contrast-enhanced computed tomography, the ampullary tumor was not visible, but the main pancreatic duct within pancreatic head and isthmus was dilated (indirect radiological tumor signs). An absence of the celiac trunk (CT) was established via computed tomography. Therefore, computed tomography-based angiography (angio-CT) of the abdominal aorta (AA) was performed before operation.

**Results:**

Angio-CT confirmed an extremely rare vascular anomaly: an absence of CT. The left gastric (LGA), splenic (SA), and common hepatic (CHA) arteries connected above origin of the superior mesenteric artery (SMA) from the AA. Pylorus-preserving pancreaticoduodenectomy (PD) was performed. This anomaly was also confirmed intraoperatively. The postoperative course was uneventful and the patient was discharged on postoperative day 10. There were no signs of recurrence of the tumor during the 6 months follow-up.

**Conclusion:**

The proper preoperative identification of anomalies within major abdominal vessels and its relationship to the tumor is very important to avoid intraoperative vascular injury and major postoperative complications.

## Case report

**Fig. 1:**
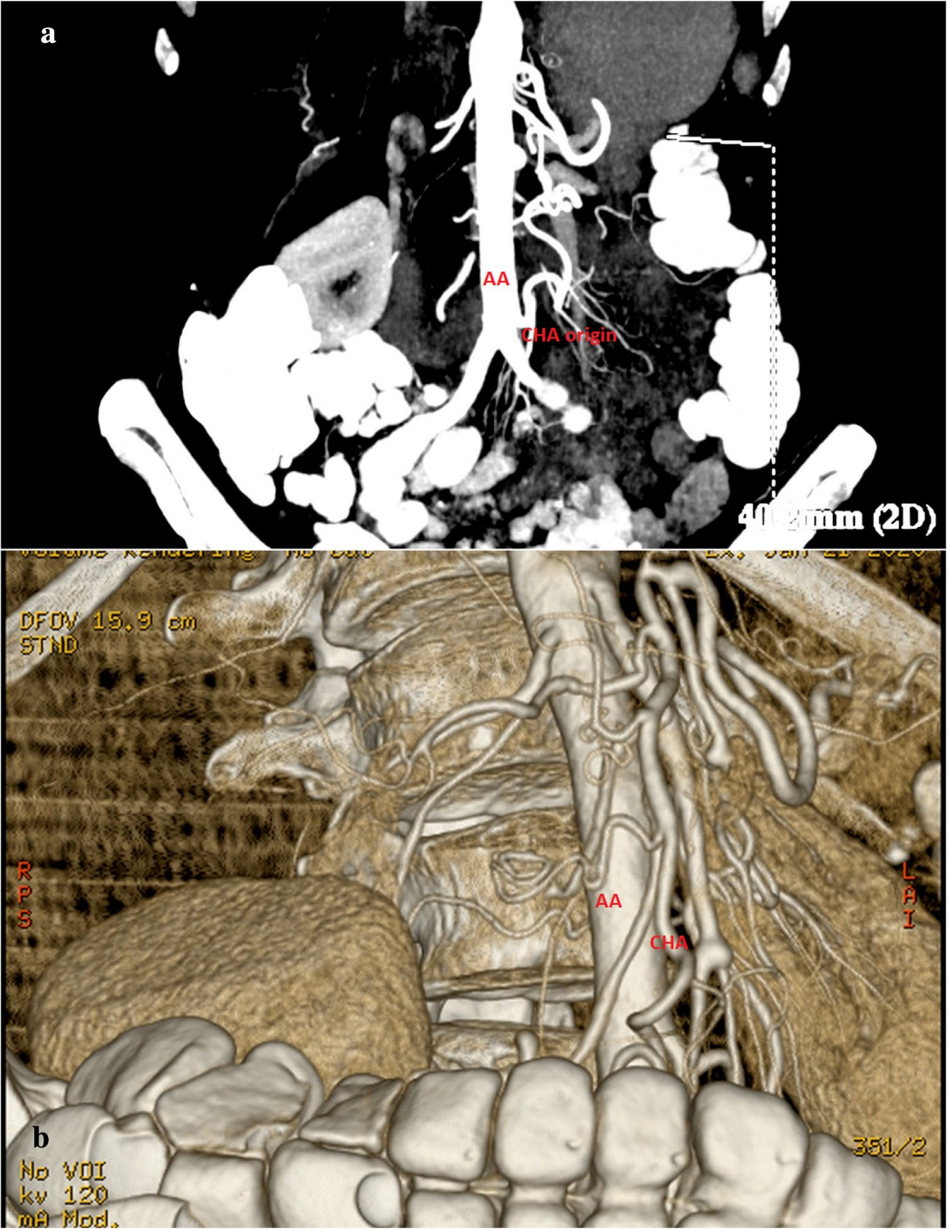
Computed tomography angiography (**a**) with reconstruction (**b**). An absence of CT. The left gastric (LGA), splenic (SA), and common hepatic (CHA) arteries connected above origin of the superior mesenteric artery (SMA) from the AA. SA and CHA filled with collateral circulation from the superior and inferior mesenteric arteries—a network of small vessels in the retroperitoneal space and at the head of the pancreas was visible. CHA arose directly from the anterior wall of abdominal aorta (AA) just above aortic bifurcation. The right hepatic artery (RHA) arose from the proximal CHA

A 77-years-old woman was admitted to our department for surgical treatment of ampullary adenocarcinoma G2 confirmed in endoscopic retrograde cholangiopancreatography (ERCP) with papillotomy and ampullary biopsy. Thirty months prior to the admission the following symptoms had occurred: abdominal pain, vomitus and cholestasis. In the contrast-enhanced computed tomography, the ampullary tumor was not visible, but the main pancreatic duct within pancreatic head and isthmus was dilated (indirect radiological tumor signs). An absence of the celiac trunk (CT) was established via computed tomography. Therefore, computed tomography-based angiography of the abdominal aorta (AA) was performed before operation. It confirmed an extremely rare vascular anomaly: an absence of CT. The left gastric (LGA), splenic (SA), and common hepatic (CHA) arteries connected above origin of the superior mesenteric artery (SMA) from the AA (Fig. 1).

The technical parameters of CT were as follows: device: CT Light Speed Pro32. CT was performed helically, the lamp perpendicular to the table (the angle tilt 0), table feed per rotation 27.5, gantry time rotation 0.4 s, slice thickness 0.625 mm = single collimation, that is means the examination was performed in the “Aortic” protocol, slice thickness 0.625 mm, total collimation with 20, KVP 120, mAs automatic matching, maximal value 594, Body filter, Pitch 1.375; matrix 512 × 512; field of view 36.

The examination was performed using a spiral technique, before and after the intravenous administration of 90 ml of Omnipaque 350.

Physical examination of the abdomen was unremarkable without any pathological mass. In subsequent laboratory investigations morphology of the peripheral blood, basic electrolytes concentrations, biochemical liver and pancreatic parameters were within the normal limits.

**Fig. 2:**
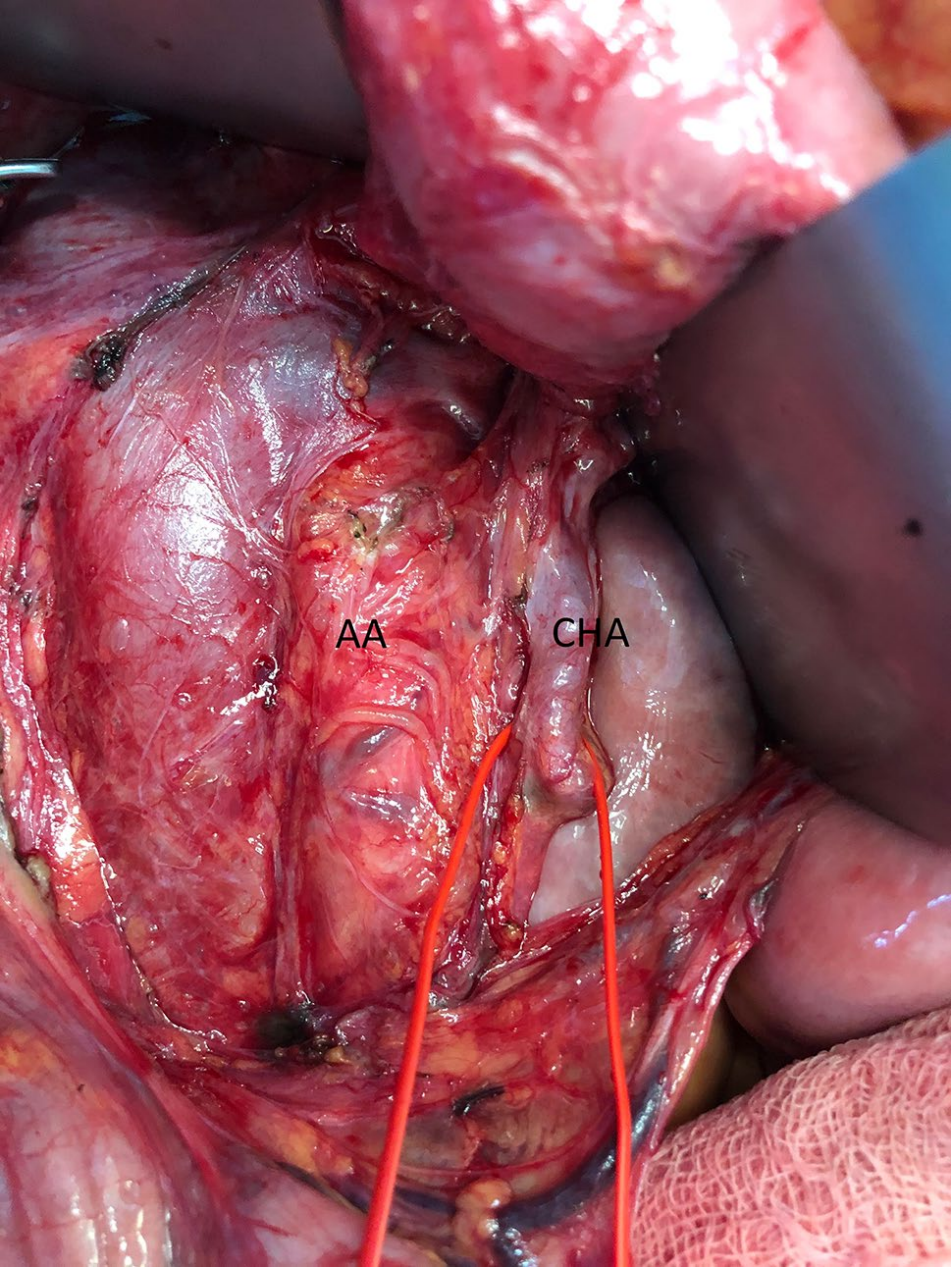
Intraoperative picture. The inferior vena cava, left renal vein were dissected and the abdominal aorta exposed. On the other left mesocolon side, an arterial vessel—common hepatic artery (CHA)—was dissected just above aortic bifurcation. It arose from AA and had unusual ascending course leading to the liver

Pylorus-preserving pancreaticoduodenectomy (PD) was performed. “Artery first” approach to PD was used [2]. Intraoperatively: arterial vessels to the liver within the hepatic-duodenal ligament were carefully dissected. The inferior vena cava, left renal vein were dissected and the abdominal aorta exposed. Medial uncinate approach has been applied for complete dissection of the superior mesenteric vein (SMV) and SMA. On the other left mesocolon side, an arterial vessel was dissected just above aortic bifurcation. It revealed unusual ascending course leading to the liver–CHA (Fig. 2). A gastro-duodenal artery (GDA) was dissected and ligated. During dissection of the aforementioned CHA, the pulse on the hepatic arteries was repeatedly monitored before closing the vessels surrounding the pancreatic head and before closing and ligating GDA. The postoperative course was uneventful and the patient was discharged on postoperative day 10. There were no signs of recurrence of the tumor during the 6 months follow-up.

## Discussion

Trifurcation into three main branches LGA, SA and CHA is the classical most common configuration of the CT. It was described by Haller and called tripus Halleri in 1756. In 1928, Adachi’s classification of the CT was introduced. It distinguished six main types of divisions of the CT and SMA. In Adachi’s classification, absence of the CT with direct origins of all its branches from aorta was not described. Only the gastro splenic trunk (as type VI in Adachi’s classification) was reported in 3% of studied population. The absence of the CT was described in Morita’s classification as type V and Uflacker’s classification as type VIII [1, 3]. In 1966, the Michel’s classification presented variations of the hepatic arteries [1]. The independent origin of CHA from AA was not reported in this classification [3].

We present an absence of the CT with direct origins of three main branches from the anterior wall of the AA. It should be emphasized that CHA arose just above aortic bifurcation.

Various variations of the CT develop during embryogenesis. An embryological explanation for the variations of CT and SMA was performed by Tandler. During embryogenesis, the roots of the ventral segmental arteries are united by a “longitudinal anastomosis”. CT is formed by the fusion of first three roots and gets separated from the fourth root. Agenesis of CT could may be caused by failure of the fusion of the first three roots. Persistence of three independent roots may give rise to the three branches of CT that originate separately from the AA at different levels [1, 3].

The most frequently (over 70% of cases), CT arises from AA at the level of inter-vertebral disc between T12 and L1 [3]. In our patient, there was a very low origin of CHA, just above aortic bifurcation (at the level L4). The mean rate of an absent CT is only 0.4% [3]. According to our knowledge, such low origin of CHA has been not described in the worldwide literature. The described here occurrence is probably the first such case reported in literature. Also, a successful radical PD in a patient with this anomaly has not been previously reported in the worldwide medical literature.

Our case presents not only a new, not described and not classified vascular anatomy, but also a successful radical complex pancreatic resection in this patient.
